# Oviposition Deterrent and Larvicidal and Pupaecidal Activity of Seven Essential Oils and their Major Components against *Culex quinquefasciatus* Say (Diptera: *Culicidae*): Synergism–antagonism Effects

**DOI:** 10.3390/insects9010025

**Published:** 2018-02-14

**Authors:** Sergio Andrade-Ochoa, Daniela Sánchez-Aldana, Karla Fabiola Chacón-Vargas, Blanca E. Rivera-Chavira, Luvia E. Sánchez-Torres, Alejandro D. Camacho, Benjamín Nogueda-Torres, Guadalupe Virginia Nevárez-Moorillón

**Affiliations:** 1 Facultad de Ciencias Químicas, Universidad Autónoma de Chihuahua, Circuito Universitario S/N, Campus Universitario II. Chihuahua, Chihuahua 31170, Chihuahua, México; s.andrade.rat@gmail.com (S.A.-O.); bchavira@uach.mx (B.E.R.-C.); 2 Escuela Nacional de Ciencias Biológicas, Instituto Politécnico Nacional. Prolongación de Carpio y Plan de Ayala S/N. Col. Santo Tomas 11340, México, DF, México; faby.chv@gmail.com (K.F.C.-V.); luviasanchez@hotmail.com (L.E.S.-T.); adcamachov18@gmail.com (A.D.C.); bnogueda@gmail.com (B.N.-T.); 3 Departamento de Investigación de Alimentos. Facultad de Ciencias Químicas. Universidad Autónoma de Coahuila, Saltillo 25000, Coahuila, México; danielasav9@gmail.com

**Keywords:** Essential oils, disease vector mosquitoes, larvicidal activity, repellency, terpenes, synergism effects, *Culex quinquefasciatus*

## Abstract

The larvicidal activity of essential oils cinnamon (*Cinnamomum verum* J. Presl), Mexican lime (*Citrus aurantifolia* Swingle) cumin (*Cuminum cyminum* Linnaeus), clove (*Syzygium aromaticum* (L.) Merr. & L.M.Perry), laurel (*Laurus nobilis* Linnaeus), Mexican oregano (*Lippia berlandieri* Schauer) and anise (*Pimpinella anisum* Linnaeus)) and their major components are tested against larvae and pupae of *Culex quinquefasciatus* Say. Third instar larvae and pupae are used for determination of lethality and mortality. Essential oils with more than 90% mortality after a 30-min treatment are evaluated at different time intervals. Of the essential oils tested, anise and Mexican oregano are effective against larvae, with a median lethal concentration (LC_50_) of 4.7 and 6.5 µg/mL, respectively. Anise essential oil and *t*-anethole are effective against pupae, with LC_50_ values of 102 and 48.7 µg/mL, respectively. Oregano essential oil and carvacrol also have relevant activities. A kinetic analysis of the larvicidal activity, the oviposition deterrent effect and assays of the effects of the binary mixtures of chemical components are undertaken. Results show that anethole has synergistic effects with other constituents. This same effect is observed for carvacrol and thymol. Limonene shows antagonistic effect with *β*-pinene. The high larvicidal and pupaecidal activities of essential oils and its components demonstrate that they can be potential substitutes for chemical compounds used in mosquitoes control programs.

## 1. Introduction

A large proportion of human population is exposed to infectious diseases spread by mosquitoes, including *Culex* spp., *Anopheles* spp. and *Aedes* spp., which are vectors of parasitic diseases [1]. *Culex quinquefasciatus* Say is a vector of lymphatic filariasis. The disease is widely distributed in tropical regions, with around 150 million people infected, ranking filariasis as one of the main causes of global morbidity [2]. *Culex quinquefasciatus* is present in tropical areas, a household pest in many urban settings, and widely reported as a vector resistant to registered insecticides [3].

Tropical areas are more prone to vector-borne parasitic diseases and the risk has increased due to the intensification of globalization [4]. Changes in climatic conditions due to global warming have promoted favorable conditions (temperature and humidity) for the development of mosquito populations, including hematophagous species. Poor sanitary conditions for water storage intended to household use, as well as unsanitary management of urban solid waste, can increase mosquito populations in urban and rural areas [1].

On the other hand, the intensive use of synthetic insecticides in mosquito control programs has created resistance in the insect population [5], undesirable effects on other insects [6] and negative impacts on the environment [7]. Although the use of insecticide is the main method in the global effort for mosquito control [8], vector-borne diseases are persistent due to mosquitoes resistant to traditionally used insecticides [9] and lack of effective vaccines [10]. The resistance of mosquitoes to synthetic insecticides has led to an interest in natural products with potential insecticidal activity, especially those derived from plants, which are considered valid alternatives to conventional synthetic pesticides to control a variety of insect pests and vectors. In this context, the essential oils and their constituents have received much attention as potentially useful bioactive compounds against insects [11]. The complex and variable mixtures of bioactive compounds with different modes of action, offered by plants, may lessen the chance of resistance in mosquito populations [12].

Essential oils are heterogeneous mixtures of organic chemical compounds from different chemical families [13]; many have a terpenoid backbone, especially monoterpenes and sesquiterpenes. Low molecular weight aliphatic compounds, acyclic esters or lactones may also be present [14]. Chemical composition of essential oils is affected by factors such as plant species and subspecies, geographical location, harvest time, part of the plant used and the extraction methods used to obtain the essential oil [15]. Several studies have reported the larvicidal action of medicinal plants and their essential oils against insect vectors of *Culex* and *Anopheles* genera, as well as *Aedes aegypti* (L.) [16,17]. Furthermore, there are many secondary metabolites found in most of those plants that are considered to have insecticidal properties [18,19].

The aim of this paper is to determine, by laboratory bioassays, the larvicidal activity of seven essential oils on third instar larva and pupa of *C. quinquefasciatus.* Essential oils tested include cinnamon (*Cinnamomum verum* J. Presl), Mexican lime (*Citrus aurantifolia* Swingle) cumin (*Cuminum cyminum* Linnaeus), clove (*Syzygium aromaticum* (L.) Merr. & L.M. Perry), laurel (*Laurus nobilis* Linnaeus), Mexican oregano (*Lippia berlandieri* Schauer) and anise (*Pimpinella anisum* Linnaeus)*.* Larvicidal effect and synergistic behavior of the major components present in the essential oils included in the study are also tested.

## 2. Materials and Methods

### 2.1. Insect Cultures and Rearing Conditions

Larvae of *C. quinquefasciatus* were collected from water tanks in the Sanctorum Cemetery in Mexico City, Mexico (19°27′17″ N, 99°12′47″ W). Identification of adults and larvae was done based on Harwood and James descriptions [20]. Mosquitoes larvae were pooled into groups of 50 individuals of first and second instars in glass bottles containing distilled water. Afterwards, larvae were maintained at 26 ± 2 °C with a natural photoperiod and supplied with powdered mixture of dog food and baking powder (3:1). The third instar emerging larvae were then separated into groups of 10 individuals in 100 mL tubes containing distilled water.

### 2.2. Plant material

For the vegetative materials used in this study, cinnamon cortex (*Cinnamomum verum*), cumin leaves (*Cuminum cyminum*), clove bottoms (*Syzygium aromaticum*), laurel leaves (*Laurus nobilis*), Mexican oregano leaves (*Lippia berlandieri*) and anise seeds (*Pimpinella anisum*) were purchased as commercial spices from Commercial Cardona S. A. (Chihuahua Chihuahua, México). Mexican lime (*Citrus aurantifolia*) bagasse was obtained from fruits brought at a local supermarket. Pure chemical compounds identified as major components of the essential oils were purchased from a Sigma-Aldrich (San Louis, MI, USA) distributor.

### 2.3. Essential Oils Extraction and Characterization

A modified Schilcher apparatus was used for hydrodistillation; 200 g of plant material (dried and chopped) was added to 4 L of water and introduced into the boiling flask and the system was heated at 100 °C for 5 h [21,22]; for Mexican lime essential oil, 400 g of fresh lime bagasse and 4 L of water were used and the system was heated at 100 °C for 4 h (Aldana et al. 2014 [23]); the essential oil extracted was dried over anhydrous sodium sulfate and stored at 4 °C in amber glass vials. For chemical analysis, conditions were similar to those reported by Aldana et al. [23] for gas chromatographic separation followed by mass spectrometry. Analysis were done in a Perkin Elmer AUTOSYSTEM XL Gas Chromatograph (Waltham, MA, USA) and TurboMass Gold Spectrometer (Hewlett-Packard In., Palo Alto, CA, USA).

### 2.4. Bioassays and Statistical Analysis

Mosquito larvicidal assays were carried out according to standard World Health Organization (WHO) larvicidal assay method with slight modifications [24]. The essential oils and the binary mixtures of the most effective essential oils (i.e., the oils which achieved the lowest LC_50_ values on *C. quinquefasciatus* larvae) were diluted in dimethyl sulfoxide (DMSO) (Sigma-Aldrich, San Louis, MI, USA) preparing a serial dilution of test dosages. For each experimental treatment, 1 mL of serial dilution was added to 224 mL of distilled water in a 500-mL glass bowl and shaken lightly to ensure a homogenous test solution. The selected larvae were transferred in distilled water into a bowl of prepared test solution with final surface area of 125 cm^2^ (25 larvae/beaker) and maintained in starvation throughout the experimental period; the surviving larvae were counted to record larval mortality. Five replicates were run simultaneously with at least 10 dosages (300, 200, 100, 75, 50, 25, 15, 10, 5, and 2.5 µg/mL). The larvicidal activity of DMSO was also determined under the same conditions; a DMSO concentration of 1000 µg/mL had no larvicidal activity. The lethal concentrations (LC_50_ and LC_90_) were calculated using Probit analysis. Data were processed using MS Excel 2010 (Microsoft, WA, USA) and SAS v.9 (Proc Probit) (SAS Institute Inc. Cary, NC, USA) computer programs.

### 2.5. The Effect of Lethal Doses on Larval Development

Twenty-five larvae (third instar) were put into a 500-mL glass bowl containing 200 mL drinking water. Upon acclimatization (after approximately 1 h), a dose of essential oil or pure components was mixed to the water, corresponding to the calculated dose LC_50_. The essential oils and pure compounds were emulsified using DMSO; water with an adequate DMSO content was used for the control larvae. Larval mortality was observed 24 h following termination of exposure, during which time, no food was offered to the larvae. Five replicates were run simultaneously.

### 2.6. Oviposition Deterrent Effect

The oviposition deterrent test was done using the method described by Xue et al. [25]. The percent of effective repellency for was calculated using the formula:

*ER* (%) = ((*NC* − *NT*)/(*NC* + *NT*) × 100 (1)

where *ER* is the percent of effective repellency; *NC* is the number of eggs in the control sample; and *NT* is the number of eggs in treatment bowl. *ER* was determined and transformed to arcsine square root values for analysis of variance.

### 2.7. Effects of Dose and Time Period on Larvicidal Activity

Essential oils that yielded more than 90% mortality after 30 min of treatment in preliminary screening were further evaluated at different concentrations from 2.5 to 200 µg/mL at different time intervals (10 to 120 min) for the determination of LC_50_ and LC_90_ values. Larvae were considered dead if they were immobile and unable to reach the water surface [26].

### 2.8. Effects of the Binary Mixtures

Three test groups were run concurrently for each binary combination tested: the binary mixture and each of the pure compounds. The compounds were combined in a 1:1 ratio (doses LC_25_/LC_25_). The application method and experimental conditions were identical to the methods described previously in Section 2.4. Four replications of 25 larvae were tested per dose.

Actual mortalities were compared to expected mortalities based on the formula:

*E* = *O_a_* + *O_b_* (1 − *O_a_*/100) (2)

where *E* is the expected mortality and *O_a_* and *O_b_* are the observed mortalities of pure compounds at the given concentration. The factor of 100 was used to calculate the value of *E*.

The effects of mixtures were designated as either antagonistic, additive, or synergistic by analysis using *x*^2^ comparisons:

*x*^2^ = (*O_m_* − *E*)^2^/*E* (3)

where O_m_ is the observed mortality from the binary mixture and E is the expected mortality, *x*^2^ with *df*  = 1, and *p* = 0.05 is 3.84. A pair with *x*^2^ values > 3.84 and having greater than expected mortality were considered to be synergistic (or antagonistic), with *x*^2^ values < 3.84 representing additive effects [19,27].

## 3. Results

### 3.1. Essential Oils Extraction and Characterization

**Figure 1:**
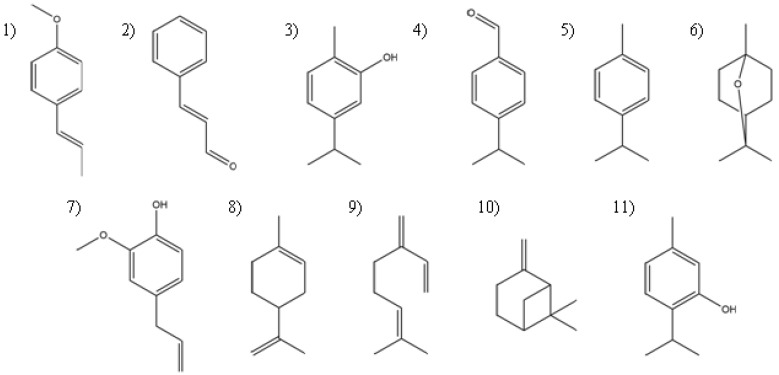
Chemical structure of major components (these are analytical standards purchased from Sigma-Aldrich as mentioned in the experimental section) evaluated: (**1**) trans-Anethole; (**2**) Cinnamaldehyde; (**3**) Carvacrol; (**4**) Cuminaldehyde; (**5**) p-Cymene; (**6**) Eucalyptol; (**7**) Eugenol; (**8**) (-)-Limonene; (**9**) β-Myrcene; (**10**) β-Pinene; and (**11**) Thymol.

Characterization of the essential oils has been reported previously by our research group [21,22,23]. Major components of each essential oil are included in Table 1 and their structures are shown in Figure 1.

### 3.2. Larvicidal and Pupaecidal Activities of Essential Oils and Their Constituents

The results of in vitro assays demonstrated the larvicidal activity of essential oils against *C. quinquefasciatus* (Diptera: *Culicidae*), since all oils tested were able to eliminated 100% of larvae at 75 µg/mL. The essential oils of Mexican oregano and anise display the highest larvicidal activity with LC_50_ of 6.21 and 4.62 µg/mL, respectively. Pupae are more resistant than larvae, since more than 100 µg/mL of essential oil were needed to reach LC_50_; the same effect has been reported before [28,29]. Table 2 and Table 3 show lethal concentrations LC_50_ and LC_90_ of the essential oils and their constituents studied against larval stage III and pupae of *C. quinquefasciatus* after 24 h exposure.

As observed, *t-*Anethole, which is the main compound of anise essential oil, proved to be efficient against larvae as well as pupae, with LC_50_ of 7.4 and 28.6 µg/mL, respectively. Carvacrol also showed efficient activity against larvae and pupae, while thymol showed lower activity, with an LC_50_ of 23.4 µg/mL against larvae and 100.5 µg/mL against pupae. Eugenol and eucalyptol show larvicidal activity with a LC_50_ of 23.04 and 24.83 µg/mL, respectively. None of the aforementioned compounds had significant activity against pupae. Percentage of *Culex quinquefasciatus* larvae mortality upon exposure to water contaminated by LC_50_ doses is shown in Table 4.

### 3.3. Oviposition Deterrent Activities of Essential Oils and Their Constituents

Oviposition deterrent activity was evaluated for the essential oils of anise, oregano and lemon, as well as its constituents, since these three essential oils were the ones with more relevant lethal potential against *C. quinquefasciatus*. As shown in Table 5, an almost 100% deterrence of female oviposition was observed for all essential oils tested in concentrations of 0.02% and 0.01%. Noteworthy differences were seen only with a concentration of 0.005%, where anise essential oil was again most efficient (repellency about 68%). Results demonstrate the high potential of these compounds to control mosquitoes; therefore, these essential oils can be considered as promising agents for the development of botanical larvicides.

### 3.4. Effect of Dose and Time on Larvicidal Activity

**Figure 2:**
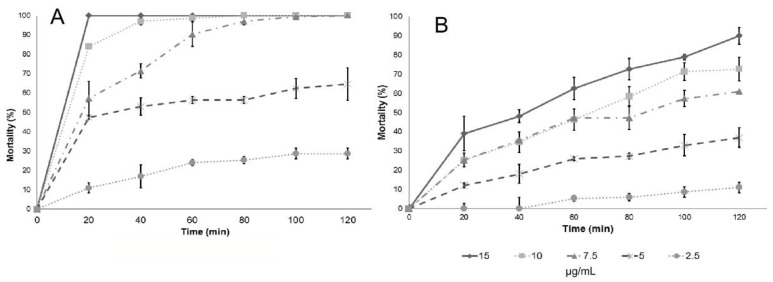
Effect of dose and incubation time on larval mortality of: (**A**) anise (*Pimpinella anisum* Linneaus) essential oil; and (**B**) *trans*-Anethole.

**Figure 3:**
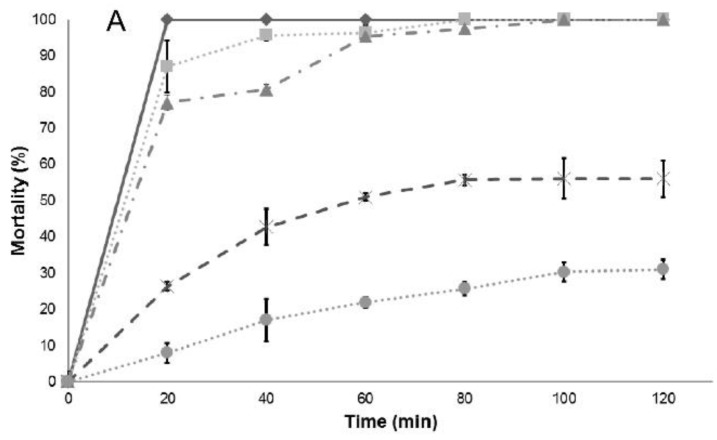

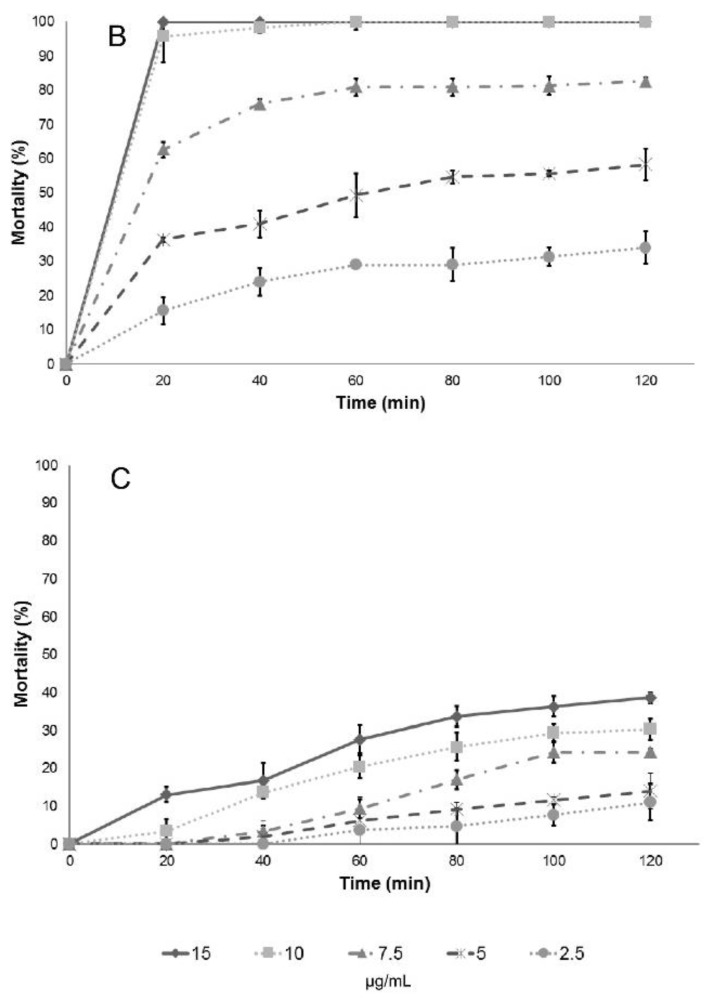
Effect of dose and incubation time on larval mortality of: *L. berlandieri* Schauer essential oils (**A**); and its major constituents: carvacrol (**B**); and thymol (**C**).

To demonstrate the effect of essential oils and their constituents on short-term exposures, dose–response kinetics was undertaken. In the present study, cases of instantaneous death were observed within 20 min of exposure. The essential oil of anise can eliminate 100% of larvae in 120 min at a concentration of 15 µg/mL, while LC_50_ is 5 µg/mL; on the other hand, *trans*-anethole, which is the major component of anise essential oil, does not remove 100% of the larvae even at concentrations higher than its LC_50_ (Figure 2). Similarly, the Mexican oregano essential oil can eliminate 100% of the larvae in 20 min at a concentration of 15 µg/mL. Similar results were obtained with carvacrol, one of its major components; however, thymol, another essential oil constituent, only eliminates 36% of larvae in 120 min (Figure 3).

### 3.5. Synergism–Antagonism Effect

The difference between the activities of the essential oils and the pure constituents, as well as the results of lethality kinetics, were the basis for the determination of synergistic/antagonistic effects among the compounds tested. If synergistic effects are found, the combination of the compounds involved can provide effective biological activity at lower concentrations [19]. In the present report, 26 binary combinations were tested against larvae (III instar) (Table 6) and pupae (Table 7), of which 20 presented a significant synergistic effect (*p*  <  0.5), while five showed no effect on mortality and one presented an antagonistic effect. *Trans*-anethole displays synergistic effects in all combinations evaluated except in combination with *β*-pinene (Table 6), while in the combination with thymol it did not present effects against the pupae (Table 7).

## 4. Discussion

Essential oils can be used as part of insecticides that can affect disease-related insect vectors, but with low impact on the accompanying insect fauna [29,30]. There are reports on essential oils with insecticidal and inhibitory oviposition capacity against *A. aegypti* [28]. The potential biological activity of the different essential oils varies according to plant species, its origin and its composition [31,32].

Regarding the larvicidal activities evaluated on instar III, there were no differences among the essential oils studied, except for the cinnamon essential oil (*C. verum* J. Presl), which has a variation on LC_50_ and LC_90_ values. Results included in Table 2 demonstrate that the essential oils tested can effectively control *C. quinquefasciatus* pupae and larvae, and the effect can be the result of the interaction of the many compounds found in each essential oil, many in small or trace quantities, or can be attributed mainly to major components. To answer the latter question, the larvicidal and pupaecidal activity of the chemical compounds reported as major constituents of the essential oils tested were analyzed, and the results are shown in Table 3. 

Lethal concentration tests (LC_50_ and LC_90_) provide information on the concentration of a given compound to decrease a population by 50% and 90%, respectively. Usually the effect is not linear; therefore, it is important to determine both values. In the determination of those values, at least three replicates are needed for each concentration in the analysis, and this is the reason for the report of confidence intervals (provided in parenthesis in Table 2 and Table 3). However, it is not possible to assess differences among the essential oil or the main components, so the analysis of larval mortality was also included in this work. The essential oils and their main components included in this study also showed a high effectiveness with respect to mortality upon exposure of *Culex quinquefasciatus* larvae to water contaminated with lethal doses (Table 4): in most cases, deaths occurred after short-term exposures.

Results of larval mortality demonstrated that the activity of anise essential oil and *trans*-anethole are statistically similar to the activity of Temephos, while *L. nobilis* L. essential oil and its main component (eucalyptol) were less effective. Cinnamaldehyde, the major component of cinnamon essential oil, showed higher LC_50_ compared to the essential oil, suggesting that there are antagonistic effects of some components present in cinnamon essential oil. On the contrary, cuminaldehyde the major constituent of cumin essential oil, is more active than the essential oil. 

Eugenol and eucalyptol, by themselves had better activity than the essential oil where they are present. In some cases, such as with eucalyptol that is present in bay laurel essential oil, the larvicidal activity doubles when the molecule is pure. This suggests that the other constituents of the essential oil have antagonistic interactions with eucalyptol. The low larvicidal activity of eucalyptol (1,8-cineol) has been reported previously against larvae of *Aedes aegypti* [33], *Anopheles anthropophagus* [34] and *Culex pipiens* [35], however it has been shown to be effective as a repellent in foods and highly effective as an ovipositional repellent [33], however the effect did not last for more than 30 min [36]. 

Limonene has a larvicidal activity similar to the essential oil of Mexican lime against stage III larvae of *C. quinquefasciatus*. Its activity doubled when the molecule was evaluated as a pure compound on pupae. This suggests that other components of the essential oil have an antagonist effect with limonene or that the compound is more effective against larvae in a more mature stage. These results demonstrate the importance of studying the components of the essential oils separately, as different studies could rule out some important molecules because the oil as a whole has seemingly unimportant biological activities. Eleni et al. [37] reported the larvicidal activity of limonene rich essential oil from *Citrus auranitium* subsp. bergamia against *Culex pipiens*; the oil displays a LC_50_ of 58.73 mg/L. On the other hand, *Mentha longifolia* essential oil, containing 20% limonene, has been reported as larvicidal against *Culex pipiens* with a LC_50_ of 78.28 mg/L after 48 h of exposure [38]. 

There are multiple plant species collectively known as Oregano, including plants from the Verbenaceae and the Lamiaceae families, that have in common the presence of thymol and carvacrol in different proportions, but with similar odor and flavor characteristics [22] and, as such, are comparable in biological activities. In this study, the essential oil of Mexican oregano (*Lippia berlandieri* Schauer) has carvacrol as its major constituent (57%), followed by thymol as the second major compound. There is no difference between the larvicidal activity of carvacrol and the essential oil of Mexican oregano, but the larvicidal activity of thymol is significantly lower. The only structural difference between carvacrol and thymol is the position of the hydroxyl group on the benzene ring with respect to the largest aliphatic chain (Figure 1); this demonstrate the importance of the aliphatic chain and its proximity to other functional groups.

There are numerous reports on the insecticidal activity of the essential oils from *Origanum* species; major components such as carvacrol, thymol, γ- terpinene and terpinen-4-ol are reported with fumigant and repellent activity rather than contact toxicity [39]. Cetin and Yanikoglu determined the insecticidal activity of essential oils from two species of Origanum (*Origanum onites L.* and *Origanum minutiflorum*) on the third and fourth instar larvae of *Culex pipiens L.*: the LC_50_ values were 24.8 and 73.8 µg/mL, respectively [40]. The authors attribute the larvicidal activity to carvacrol, as also suggested in this report. However, it is important to note that other studies have reported that oregano essential oil with high levels of thymol have an effective deterrent oviposition activity against *Culex quinquefasciatus* females [41]. 

Regarding essential oils derived from plants of the genus *Lippia*, this study is the first to report the activity of the essential oil of *Lippia berlandieri* Shauer. Vera et al. evaluated the activity of *Lippia alba* and *Lippia origanoides* against larvae of *Aedes aegypti* [42]. These two essential oils contained carvone and carvacrol as major components, and presented LC_50_ values of approximately 50 µg/mL. In addition, the essential oils of *Lippia gracilis* and *Lippia sidoides* have been evaluated against *Aedes aegypti* [43,44]. Gleiser and Zygadlo evaluated the activity of the essential oils of *Lippia turbinata* and *Lippia polystachya* against *Culex quinquefasciatus* larvae [45]. Both oils presented α-thujone as the major component and showed no relevant larvicidal activity.

The larvicidal efficiency of anise essential oil as well *trans*-anethole, has been previously reported [46]. Waliwitiya et al., Pavela and Sousa et al. evaluated the activity of *trans*-anethole against larvae of *Aedes aegypti*, *Anopheles atroparvus* and *Culex quinquefasciatus* respectively, finding relevant LC_50_ values [47,48,49]. This study confirms the activity of *Pimpinella anisum* L. essential oil, but it shows that *trans*-anethole does not eliminate the larvae as quickly as it does the essential oil, even though both have similar LC_50_ values. This suggests that there are interactions with other components that are present in the essential oil at lower or even trace concentrations. Therefore, it is always important to analyze the activity of the complete essential oil and their major components, since the presence of compounds different than the main components, can present synergistic or antagonistic effects. 

The most relevant synergistic effects are observed against larvae with the combination of *trans*-anetol and limonene and the combination of *trans*-anethole and β-myrcene against pupae. These effects may be responsible for the rapid larvicidal activity of the essential oil of *P. anisum*. Synergistic effects are also observed when mixing carvacrol and thymol, the main constituents of *Lippia berlandieri* essential oil. However, none of these compounds has additive effects on the activity when they are mixed with *p*-cymene. The only antagonistic effect was observed with the limonene–pinene combination, which may be related to the difference in activity between *C.aurantifolia* essential oil and limonene against pupae.

The mechanism of action with which the essential oils exert their larvicidal activity is not completely described. Pratti et al. suggest that instant death is due to severe damage of Malpighian tubules, since they are responsible for the excretion, not only of electrolytes and metabolites, but also of the high volume of water naturally present in insect larvae environment [50]. There are reports suggesting that spice essential oils have neurotoxic poisoning-type effect on insects, similar to the ones produced by organophosphate and carbamate insecticides, by inhibition of the of the acetylcholinesterase enzyme (AChE) [51]. In a comparative study of the vapor action of essential oils from plants of the Lamiaceae family on *R. dominica* adults, it was observed that the essential oils inhibited about 65% of AChE activity, while limonene lowered only 2% of the enzymatic activity [52]. Furthermore, these authors found that the essential oils significantly increased the levels of cyclic adenosine monophosphate (AMP) (even at very low concentrations), which suggests a possible action on octopamine. Similar results were obtained by Enan in flies and cockroaches exposed to eugenol and α-terpineol [53]. In silico studies have demonstrated that terpenes can interact with AChE of *A. aegypti*, by joining a hydrophobic site of the enzyme, with an interaction with glycine 412, 409, abd 412 and isoleucine 413 amino acids [54]. Another proposed mechanism of action has been presented by Priestley et al., who suggest that thymol acts on GABA receptors of *Drosophila melanogaster* [55]. 

## 5. Conclusions

In conclusion, the high larvicidal activity of essential oils and their constituent compounds make them potential substitutes for traditionally used chemical compounds in larval and pupal stages of mosquito control programs. The essential oil is readily available and the cost constraint can be overcome by the low value of the LC_50_. The larvicidal activity of Essential oils shows that it is not necessary to use the pure active compound, since the complex mixture of compounds present in essential oils are effective as larvicide.

On the other hand, their principal constituents are low molecular weight compounds and therefore easily synthesized and the study of the pure components is essential to elucidate the larvicidal and insecticidal mechanism of action of essential oils and their constituents.

## Figures and Tables

**Table 1:** Main essential oil components of the seven essential oils investigated for larvicidal toxicity against the filariasis vector *Culex quinquefasciatus*.

Essential Oil	Major components
*Cinnamomum verum* J. Presl	Cinnamaldehyde (98.3%), 3-carene (0.3%), β-caryophyllene (0.1%)
*Citrus aurantifolia* Swingle	Limonene (98.6%), *β*-pinene (0.1%), *α*-pinene (0.1%)
*Cuminum cyminum* Linnaeus	Cuminaldehyde (89.6%), *α*-pinene (3.2%), limonene (1.1%)
*Syzygium aromaticum* (L.) Merr. & L.M. Perry	Eugenol (96.3%), chavicol (1.23%), β-caryophyllene (1.1%)
*Laurus nobilis* Linnaeus	Eucalyptol (76.1%), *α*-pinene (5.7%) β-myrcene (4.6%)
*Lippia berlandieri* Shauer	Carvacrol (57.5%), thymol (32.8%), *p*-cymene (1.8%)
*Pimpinella anisum* Linnaeus	t-Anethole (78.0%), β-myrcene (15.3%), limonene (2.1%)

**Table 2:** LC_50_ and LC_90_ (µg/mL) of essential oils on *Culex quinquefasciatus* at III instar and pupal stages after 24 h of exposure.

Essential Oils	III Instar			Pupae		
	LC_50_	LC_90_	Chi **	LC_50_	LC_90_	Chi **
*Cinnamomum verum* J. Presl	24.5 (23.2–25.8)	53.7 (52.2–55.1)	1.198	216.7 (212.8–220.6)	374.1 (359.8–388.3)	0.055
*Citrus aurantifolia* Swingle	14.1 (13.9–14.3)	30.9 (28.4–33.4)	0.039	184.9 (180.0–189.7)	381.0 (365.6–396.5)	1.331
*Cuminum cyminum* Linnaeus	23.3 (21.8–24.9)	56.3 (52.4–60.1)	0.048	98.4 (95.9–101.3)	212.6 (199.5–225.8)	0.020
*Syzygium aromaticum* (L.) Merr. & L.M. Perry	22.5 (21.5–23.7)	49.7 (47.0–52.4)	1.326	236.5 (232.5–240.4)	447.0 (433.7–460.3)	1.202
*Laurus nobilis* Linnaeus	49.0 (48.5–50.5)	111.0 (107.0–115.0)	0.674	204.9 (201.8–208.1)	460.1 (442.4–447.8)	0.571
*Lippia berlandieri* Shauer	6.5 (5.9–6.9)	18.8 (17.8–19.8)	0.268	181.0 (178.4–183.6)	356.1 (341.0–371.1)	0.645
*Pimpinella anisum* Linnaeus	4.6 (3.5–5.8)	9.0 (8.5–9.5)	2.572	51.6 (49.2–54.0)	102.0 (85.1–118.8)	0.016
Temephos H (control)	2.1 (1.8–2.5)	5.2 (4.1–6.4)	0.039	34.0 (29.1–39.0)	49.2 (43.4–54.9)	0.048

In parenthesis, 95% confidence intervals, essential oils activity is considered significantly different when the 95% CI fail to overlap. ** Chi-square value, significant at *p* < 0.05 level.

**Table 3:** LC_50_ and LC_90_ (µg/mL) of the major constituents of the essential oils at III instar and pupal of *Culex quinquefasciatus* after 24 h of exposure.

Compounds	III Instar			Pupae		
	LC_50_	LC_ 90_	Chi **	LC_ 50_	LC_ 90_	Chi **
*t*-anethole	7.4 (6.5–8.3)	18.8 (17.4–20.2)	0.055	28.6 (26.4–30.8)	48.6 (45.79–51.51)	0.039
carvacrol	5.5 (5.2–5.7)	11.3 (9.9–2.7)	2.684	53.2 (49.9–56.6)	111.4 (102.0–120.8)	0.483
cinnamaldehyde	18.4 (17.6–19.2)	39.2 (35.2–43.1)	1.203	90.1 (84.6–95.6)	179.3 (170.4–188.1)	0.048
cuminaldehyde	23.0 (21.9–24.1)	52.2 (50.1–54.3)	4.313	95.4 (91.1–99.6)	198.2 (193.7–202.7)	0.896
*p-*cymene	23.1 (21.2–24.9)	91.9 (89.7–94.0)	0.253	306.3(297.4–315.1	549.2 (533.0–565.3)	1.326
eucalyptol	24.8 (22.7–26.8)	48.0 (44.9–51.1)	0.116	92.9 (89.1–96.6)	193.9 (186.9–201.0)	0.574
eugenol	23.0 (21.7–24.3)	51.2 (46.6–55.8)	0.279	106.9(99.5–114.3)	198.4 (189.6–207.1)	0.665
(-)-limonene	14.2 (13.5–15.0)	36.4 (33.5–39.4)	0.572	78.4 (73.3–83.4)	155.0 (145.6–164.3)	0.865
myrcene	19.5 (18.5–20.4)	40.0 (36.7–43.2)	2.561	31.8 (30.4–33.1)	59.1 (55.2–62.9)	1.287
*β*-pinene	19.6 (18.8–20.3)	61.9 (57.8–65.9)	0.265	206.9(95.3–105.8)	458.4 (440.1–476.6)	3.404
thymol	23.4 (22.5–24.4)	45.4 (43.3–47.6)	0.683	100.57 (88.3–111.8)	168.7 (155.8–181.6)	1.642

In parenthesis, 95% confidence intervals, essential oils activity is considered significantly different when the 95% CI fail to overlap. ** Chi-square value, significant at *p* < 0.05 level.

**Table 4:** Percentage of *Culex quinquefasciatus* larvae mortality upon exposure to water contaminated by LC_50_ doses.

Essential Oils	Larval Mortality	Compounds	Larval Mortality
*Cinnamomum verum* J. Presl	52.7 ± 7.6 ^a^	cinnamaldehyde	62.3 ± 9.1 ^a^
*Citrus aurantifolia* Swingle	54.7 ± 5.8 ^abc^	(-)-limonene	56.2 ± 9.7 ^ab^
		*β*-pinene	49.6 ± 7.3 ^ab^
*Cuminum cyminum* Linnaeus	52.2 ± 0.7 ^abc^	cuminaldehyde	56.2 ± 11.7 ^ab^
*Syzygium aromaticum* (L.) Merr. & L.M. Perry	52.9 ± 4.9 ^abc^	eugenol	56.7 ± 7.4 ^ab^
*Laurus nobilis* Linnaeus	65.3 ± 10.9 ^a^	eucalyptol	63.8 ± 4.8 ^a^
*Lippia berlandieri* Shauer	60.7 ± 5.8 ^ab^	carvacrol	58.7 ± 6.6 ^ab^
		thymol	48.7 ± 3.4 ^ab^
*Pimpinella anisum* Linnaeus	48.2 ± 6.1 ^bc^	*t*-anethole	47.1 ± 5.8 ^ab^
		myrcene	58.7 ± 8.3 ^ab^
Temephos H (control)	46.8 ± 6.5 ^c^	Temephos H (control)	43.3 ± 7.6 ^b^
Control (Water)	0.0 ± 0.0	Control (Water)	0.0 ± 0.0

Numbers in column followed by different letters are significantly different at level of *p* < 0.05 according to Tukey’s test.

**Table 5:** Oviposition deterrent activity of *P. anisum, L. berlandieri* and *C.aurantifolia* essential oils and their major components against gravid female *Culex quinquefasciatus.*

	Effective repellency (%)						
Concentration (%)	*P. anisum* Essential Oil	Anethole	*L. berlandieri* Essential Oil	Thymol	Carvacrol	*C. aurantifolia* Essential Oil	Limonene
0.02	100 ± 0.0 ^a^	100 ± 0.0 ^a^	100 ± 0.0 ^a^	100 ± 0.0 ^a^	100 ± 0.0 ^a^	100 ± 0.0 ^a^	100 ± 0.0 ^a^
0.01	100 ± 0.0 ^a^	95.2 ± 4.5 ^b^	100 ± 0.0 ^a^	87.5 ± 9.5 ^b^	100 ± 0.0 ^a^	99.3 ± 0.9 ^a^	89.1 ± 8.5 ^b^
0.005	68.6 ± 4.4 ^b^	49.0 ± 5.2 ^c^	38.0 ± 6.3 ^b^	18.9 ± 6.3 ^c^	41.3 ± 2.5 ^b^	23.6 ± 1.8 ^b^	17.6 ± 3.3 ^c^

Each value represents the mean (*±SE*) of five values. Values with different letters are significantly different at *p* < 0.05 level (Tukey’s test of multiple comparison).

**Table 6:** Effect of binary mixtures of individual compounds on the mortality against *Culex quinquefasciatus* larvae.

Compounds		Larval Mortality (%)				*x*^2^	Effect
		Pure Compounds		Binary Mixtures			
Compound A	Compound B	Observed A	Observed B	Expected	Observed		
*t*-anethole	carvacrol	13.2	27.8	35.6	86.4	72.5	Synergistic
*t*-anethole	cinnamaldehyde	13.2	8.3	18.7	71.1	147.3	Synergistic
*t*-anethole	*p*-cymene	13.2	3.2	14.2	87.6	378.1	Synergistic
*t*-anethole	eugenol	13.2	8.1	18.5	56.5	78.2	Synergistic
*t*-anethole	(-)-limonene	13.2	6.2	16.8	98.3	394.1	Synergistic
*t*-anethole	myrcene	13.2	3.3	14.3	62.7	163.4	Synergistic
*t*-anethole	*β*-pinene	13.2	5.3	16.1	15.4	0.0	No effect
*t*-anethole	thymol	13.2	12.6	22.4	74.7	122.2	Synergistic
(-)-limonene	carvacrol	6.2	27.8	31.9	57.4	20.4	Synergistic
(-)-limonene	cinnamaldehyde	6.2	8.3	13.6	42.7	62.3	Synergistic
(-)-limonene	*p*-cymene	6.2	3.2	8.8	41.2	118.9	Synergistic
(-)-limonene	eugenol	6.2	8.1	13.4	49.5	97.1	Synergistic
(-)-limonene	myrcene	6.2	3.3	8.9	31.8	58.8	Synergistic
(-)-limonene	*β*-pinene	6.2	5.3	10.8	2.1	7.0	Antagonistic
(-)-limonene	thymol	6.2	12.6	17.6	16.3	0.1	No effect
carvacrol	cinnamaldehyde	27.8	8.3	26.1	38.1	5.6	Synergistic
carvacrol	*p*-cymene	27.8	3.2	22.4	28.5	1.7	No effect
carvacrol	eugenol	27.8	8.1	25.9	81.3	118.3	Synergistic
carvacrol	myrcene	27.8	3.3	22.5	85.5	177.0	Synergistic
carvacrol	*β*-pinene	27.8	5.3	23.9	61.2	58.2	Synergistic
carvacrol	thymol	27.8	8.3	26.1	72.6	83.1	Synergistic
thymol	cinnamaldehyde	12.6	8.3	18.3	49.7	54.1	Synergistic
thymol	*p*-cymene	12.6	3.2	13.8	14.4	0.0	No effect
thymol	eugenol	12.6	8.1	18.1	67.8	136.6	Synergistic
thymol	myrcene	12.6	3.3	13.9	81.6	329.8	Synergistic
thymol	*β*-pinene	12.6	5.3	15.6	15.1	0.0	No effect

**Table 7:** Effect of binary mixtures of individual compounds on the mortality against *Culex quinquefasciatus* pupae.

Compounds		Pupae Mortality (%)				*x*^2^	Effect
		Pure Compounds		Binary Mixtures			
Compound A	Compound B	Observed A	Observed B	Expected	Observed		
*t*-anethole	carvacrol	20.3	15.7	28.7	68.4	55.0	Synergistic
*t*-anethole	cinnamaldehyde	20.3	8.3	22.8	60.2	61.4	Synergistic
*t*-anethole	*p*-cymene	20.3	2.5	18.2	66.4	128.0	Synergistic
*t*-anethole	eugenol	20.3	2.8	18.4	40.3	26.0	Synergistic
*t*-anethole	(-)-limonene	20.3	11.2	25.1	68.6	75.4	Synergistic
*t*-anethole	myrcene	20.3	18.6	31.0	80.5	79.0	Synergistic
*t*-anethole	*β*-pinene	20.3	2.7	18.3	21.2	0.4	No effect
*t*-anethole	thymol	20.3	3.1	18.6	22.3	0.7	No effect
(-)-limonene	carvacrol	11.2	15.7	23.9	42.3	14.2	Synergistic
(-)-limonene	cinnamaldehyde	11.2	8.3	17.3	37.5	23.5	Synergistic
(-)-limonene	*p*-cymene	11.2	2.5	12.2	49.3	113.3	Synergistic
(-)-limonene	eugenol	11.2	2.8	12.4	36.4	46.2	Synergistic
(-)-limonene	myrcene	11.2	18.6	26.5	61.3	45.9	Synergistic
(-)-limonene	*β*-pinene	11.2	2.7	12.3	41.3	67.9	Antagonistic
(-)-limonene	thymol	11.2	3.1	12.7	11.3	0.2	No effect
carvacrol	cinnamaldehyde	15.7	8.3	20.2	21.3	0.1	No effect
carvacrol	*p*-cymene	15.7	2.5	15.3	16.4	0.1	No effect
carvacrol	eugenol	15.7	2.8	15.6	48.7	70.3	Synergistic
carvacrol	myrcene	15.7	18.6	28.9	71.3	62.1	Synergistic
carvacrol	*β*-pinene	15.7	2.7	15.5	36.6	28.7	Synergistic
carvacrol	thymol	15.7	3.1	15.8	56.4	103.8	Synergistic
thymol	cinnamaldehyde	3.1	8.3	11.0	36.1	56.8	Synergistic
thymol	*p*-cymene	3.1	2.5	5.4	4.6	0.1	No effect
thymol	eugenol	3.1	2.8	5.7	34.8	147.9	Synergistic
thymol	myrcene	3.1	18.6	21.0	58.7	67.5	Synergistic
thymol	*β*-pinene	3.1	2.7	5.6	3.5	0.8	No effect
